# A Safe Vaccine (DV-STM-07) against *Salmonella* Infection Prevents Abortion and Confers Protective Immunity to the Pregnant and New Born Mice

**DOI:** 10.1371/journal.pone.0009139

**Published:** 2010-02-10

**Authors:** Vidya Devi Negi, Arvindhan G. Nagarajan, Dipshikha Chakravortty

**Affiliations:** Centre for Infectious Disease Research and Biosafety Laboratories, Department of Microbiology and Cell Biology, Indian Institute of Science, Bangalore, India; Columbia University, United States of America

## Abstract

Pregnancy is a transient immuno-compromised condition which has evolved to avoid the immune rejection of the fetus by the maternal immune system. The altered immune response of the pregnant female leads to increased susceptibility to invading pathogens, resulting in abortion and congenital defects of the fetus and a subnormal response to vaccination. Active vaccination during pregnancy may lead to abortion induced by heightened cell mediated immune response. In this study, we have administered the highly attenuated vaccine strain *ΔpmrG-HM-D* (DV-STM-07) in female mice before the onset of pregnancy and followed the immune reaction against challenge with virulent S. Typhimurium in pregnant mice. Here we demonstrate that DV-STM-07 vaccine gives protection against *Salmonella* in pregnant mice and also prevents *Salmonella* induced abortion. This protection is conferred by directing the immune response towards Th2 activation and Th1 suppression. The low Th1 response prevents abortion. The use of live attenuated vaccine just before pregnancy carries the risk of transmission to the fetus. We have shown that this vaccine is safe as the vaccine strain is quickly eliminated from the mother and is not transmitted to the fetus. This vaccine also confers immunity to the new born mice of vaccinated mothers. Since there is no evidence of the vaccine candidate reaching the new born mice, we hypothesize that it may be due to trans-colostral transfer of protective anti-*Salmonella* antibodies. These results suggest that our vaccine DV-STM-07 can be very useful in preventing abortion in the pregnant individuals and confer immunity to the new born. Since there are no such vaccine candidates which can be given to the new born and to the pregnant women, this vaccine holds a very bright future to combat *Salmonella* induced pregnancy loss.

## Introduction

*Salmonella enterica* is a Gram negative pathogenic bacterium which affects many warm blooded animals. *Salmonella enterica* has many serovars which have different host specificity. In humans, *Salmonella enterica* serovar Typhi (*S.* Typhi) is the causative agent of typhoid fever. S. Typhi spreads by oro-faecal route and is endemic in most of the developing countries. *Salmonella enterica* serovar Typhimurium (S. Typhimurium) had wide range of host including human being. S. Typhimurium causes gastroenteritis in human being and live stocks and systemic infection in mouse. S. Typhimurium is a major food born pathogen in developing countries [1]. Other than children, high-risk population for S. Typhi infection includes old people, pregnant women, HIV patients and transplant patient. In recent year, multidrug resistance (MDR) strains were identified in Pakistan (2005) and India (2007) [2]. *Salmonella* causes abortion in mink [3] ewes [4], cattles [5] and horse [6] which also include some recent outbreaks [7], [8], [9].

During pregnancy the immune system of mother is altered and mounts a Th2-biased response with an enhanced humoral immune response and suppressed cell-mediated immunity [10], [11]. There are many reports which show that Th1 and Th2 are related to cell mediated and humoral immunity respectively [11], [12]. This Th2-biased immune response results in increased susceptibility to certain autoimmune disease and intracellular infections and may have deleterious effect during the outcome of infection like leishmaniasis [13], malaria [14], toxoplasmosis and listeriosis [15]. Blocking placenta immunomodulatory ferritin (PLIF) decreases Th2 and increases Th1 cytokines and induces abortion [16] which highlights the importance of this Th2 biased immune response. During pregnancy along with the change in the cytokine milieu, other physiological changes including insulin resistance, thrombophilia, immunosuppression and hypervolemia, increase the risk of chronic disease [17]. The impairment of immune response during pregnancy make mothers more susceptible to rapid fatal infection by *Salmonella* [13], [18]. The exact mechanisms by which *Salmonella* infection cause abortion and impair the immune response are not clear. First case study of typhoid fever during pregnancy was reported in 1901 and was shown to result in abortion and premature birth. Then on, many such cases which shows the complications during pregnancy because of S. Typhi infection were reported [19].

Many infectious diseases which affect pregnant women can be prevented through vaccination. But vaccination in pregnant individual with live attenuated vaccine strains is always fraught with the theoretical possibility of transmission of infection to the fetus. Transmission has been shown for certain live attenuated virus like the triple vaccine for measles, mumps and rubella (MMR) and vaccinia virus [20]. The diseases for which vaccination is recommended during pregnancy by CDC's (Centers for Disease Control and Prevention) Advisory Committee for Immunization Practices (ACIP) includes hepatitis, influenza [21] (viral), pertussis, tetanus, and diphtheria (bacterial). For all the above diseases, the vaccines used are subunit, killed or toxoid vaccines which do not cause transmission of disease to the fetus and induces a Th2 mediated immune response. The two approved *Salmonella* vaccines for human are Ty21A (live attenuated) and Vi polysaccharide vaccine. The Vi antigen of *Salmonella* is encoded by highly unstable and mobile DNA island called *Salmonella* pathogenicity Island 7 (SPI-7) and the mutation has been shown in the *viaB* locus [22], [23] The isolation of Vi negative clinical isolates of S. Typhi from human patients indicates that the Vi vaccine will not protect the individuals against infection caused by Vi-negative *Salmonella* strains [24], [25], [26]. The live attenuated vaccinia virus should be given at least three months before the onset of pregnancy [20] to rule out the possibility of its transmission to the fetus. In our current vaccination regime, we have used 1 dose followed by two booster doses of our live attenuated vaccine strain Δ*pmrG-HM-D* (here after DV-STM-07); prior to mating. We found that DV-STM-07 is completely safe in terms of transmission to fetus and is effective in preventing abortion when challenged with WT during pregnancy. In our previous study, we reported that live attenuated vaccine DV-STM-07 is a very potent candidate as a vaccine and was able to reduce the bacterial load of virulent *Salmonella* from the organs of the vaccinated mice. Very low dose of vaccine is required to protect the mice from lethal dose of virulent *Salmonella* [27]. In our current study, we have tried to further elucidate the mechanism by which our vaccine strain achieves protection and at the same time prevents abortion. We have shown in this study that the natural Th2 bias of pregnancy is not altered by the vaccine strain whereas the wild type *Salmonella* induces a Th1 mediated immune response with increased Th1 cytokines (IFNγ, TNFα) leading to abortion [28], [29]. We have also shown that the protection is through a strong Th2 response. We demonstrated that the vaccine strain is able to confer immunity to the next generation. Pups of vaccinated mother were more resistant to the *Salmonella* infection than non-vaccinated mother's pups.

## Results

### Strain DV-STM-07 Is Safe for Vaccination during Pregnancy and Does Not Cause Maternal Death in High Dose

In our previous study, we have found that the minimal effective dosage of vaccine strain is 10^4^ CFU. To analyze the safety of vaccine strain during pregnancy, cohort of 6 BALB/c mice were infected orally with 10^7^ CFU of WT *Salmonella* and DV-STM-07 vaccine strain, 10-12 days post-mating. The mice were observed and the results were recorded every day post infection (Table 1). There was one maternal death in the WT infected group and none in the vaccinated group. One abortion and two cases of resorption of fetus were observed in the WT infected group. The fetal death rate was 50%. In the vaccinated group, there was only one case of resorption taking the fetal death rate to 16% and the fetuses were normal. After delivery, the pups from the control and vaccinated mothers were sacrificed and the organs were isolated, pooled and analyzed for load of *Salmonella* (WT or DV-STM-07) in different organs. Ten-twenty days old pups were sacrificed and checked for organ CFU. Heavy load of bacteria was present in the organs of the pup in case of WT infected group however, no CFU was detected in case of the vaccinated group (Data not shown). Though there was a low percentage of fetal death at dose of 10^7^ CFU/mice in case of vaccinated mice, when the vaccination dosage was reduced to 10^4^ CFU, no fetal death was observed. Hence, this dose was used in all further experiments. Statistical analysis between the different groups of vaccinated and unvaccinated mice as compared to the PBS control mice were done using Fisher's exact test. The death of pups between 10 to 20 days of birth was significantly higher in unvaccinated mice as compared to vaccinated mice.

**Table 1:** Efficacy of DV-STM-07 vaccine in pregnant mice.

Strain used	No of mice	Death of mother	Mice Aborted/resorption	No of Pups Delivered by surviving mothers	No of pups dead (0-10 Days)/Total no of pups	No of pups dead (10-20 Days)/Total no of pups
WT *Salmonella*	6	1	3	15	3/15	6/12 ( p-value = 0.004)
DV-STM-07	6	0	1	12	3/12	0/9
PBS	5	0	0	27	7/27	0/20

Pregnant BALB/c mice were infected with WT and Vaccine strain DV-STM-07 (10^7^ cfu) and were observed for abortion and the offspring outcome. With WT infection, abortion and the death of pups after delivery was more than DV-STM-07 infected mice. The data shows the representative of three independent experiments performed. Fisher's exact test was used to analyze the data. *p*-values are provided for statistically significant data.

### Vaccination with DV-STM-07 Protects Pregnant Mice from Wild Type *Salmonella* Induced Abortion and Is Not Transmitted to Fetus

The efficiency of vaccination in preventing *Salmonella* induced abortion was analyzed in BALB/c mice. The mice were vaccinated with three doses of 10^4 ^CFU/dose including two booster doses at an interval of one week each. The unvaccinated mice received PBS. Mice were mated one week after the last dose and were challenged with 10^7^ cfu of WT *Salmonella* during 13-14^th^ day of pregnancy. The course of the pregnancy till birth was monitored. There was no maternal death or fetal abortion in case of the vaccinated mice whereas in the unvaccinated group, one maternal death was observed. In the unvaccinated group, there was one case of resorption and two cases of abortion of the fetus (Table 2). The fetal death rate was 100% and the maternal death rate was 25%. Statistical analysis of data using Fisher's exact test after pooling the data under the groups death, abortion and resorption under one (unsuccessful pregnancy) against the numbers delivered (successful pregnancy) shows that the effect of vaccination in preventing fetal/maternal mortality is significant (P value = 0.0285) Apart from inducing abortion and fetal resorption, the WT bacteria also infected the new born pups. This led to high post-natal mortality of the new born pups (Table 1). The transfer of WT Salmonella to fetus raised the possibility that vaccine strain can also be transmitted to the fetus. To test this, 3 pregnant mice were inoculated orally with 10^7^ CFU of WT *Salmonella* and DV-STM-07 during mid pregnancy. One of the WT infected mother aborted. Of the remaining two wild type infected mice, one was allowed to deliver normally and the other was sacrificed 3 days post infection and the cesarean delivered pups were dissected aseptically and the pooled organs were homogenized and plated. We did not observe any bacterial growth in those pups delivered through caesarian from both WT infected and vaccinated pregnant mothers (Table 3). Further, bacteria were not present in the vaginal swab, amniotic tissue and amniotic fluid of the mice infected with vaccine strain (Table 3). However, blood and organs of WT infected pregnant mice were positive for bacteria after 3^rd^ day of infection and in one instance the amniotic fluid was also positive for the bacteria, where as the vaccine strain bacteria were completely cleared by 3^rd^ day of infection (Table 3). The absence of bacteria in the delivered pups of WT infected pregnant mice indicates that, *Salmonella* is not able to cross the trans-placental barrier when administered through oral route but neonate pups may be exposed to bacteria in the birth canal during delivery or after birth through feces or husk. Fecal shedding of WT and DV-STM-07 strain was assessed in normal mice. WT bacterial shedding was observed till day 4, but the vaccine strain was present in the fecal matter only on day 1 post infection and thereafter no shedding of the vaccine strain was observed (data not shown).

**Table 2:** Pregnancy outcome after challenging the pre-pregnancy vaccinated mice with WT.

Strain	Dose 10^4^ CFU/mouse	Mating after 1 week	Challenge With WT	Pregnancy Outcome/total pregnant mice			
			Mid pregnancy	Death (mother)	Abortion	Resorption	Delivered
DV-STM-07 Vaccine	3 doses	Mated	WT 10^7^ CFU	0/4	0/4	0/4	4/4 (P value = 0.0285)
PBS control	3 doses	Mated	WT 10^7^ CFU	¼	2/4	1/4	0/4

Vaccination with DV-STM-07 prevents abortion induced by WT challenge during pregnancy. Vaccinated (3 doses) and control mice were mated and challenged with 10^7^ CFU/mouse WT *Salmonella* during pregnancy (as described in materials and methods). There was no incidence of fetal death in vaccinated mice whereas there was one case of maternal death and two cases of abortion and one fetal resorption in unvaccinated mice. Experiment was repeated thrice to reproduce the observed results. Fisher's exact test was used to analyze the data. *p*-value = 0.0285.

**Table 3:** Transmission profile of bacteria during pregnancy in control and vaccinated mice.

Vaccination	BALB/c Pregnant (3)	CFU in Mothers Blood (X10^2^/ml)	CFU in Pups taken out by caesarian(pooled data) (X10^2^/gm wt of pup)	Growth in LB from Vaginal swab of pregnant mother	CFU in amniotic Fluid (X10^2^/ml)
WT	A	6	17.8	+	58
	B	0	0	0	0
	C	92	Dead	+	ND
DV-STM-07	A	0	0	0	0
	B	0	0	0	0
	C	0	0	0	0

Undelivered pups were dissected aseptically from WT and DV-STM-07 strain infected mice and homogenized after three days of infection. Homogenate was plated on LB agar plate and analyzed for CFU growth. There was no trans-placental transfer of *Salmonella* both in case of vaccine and WT infected mice. But WT mothers were positive for *Salmonella* in blood culture and vaginal swab culture (2 mouse) Experiment was done thrice to reproduce the results. (+; Positive, ND; Not determined).

### Cryptdine Level Was More in Vaccinated Pregnant Mice as Compared to the Control Mice

**Figure 1:**
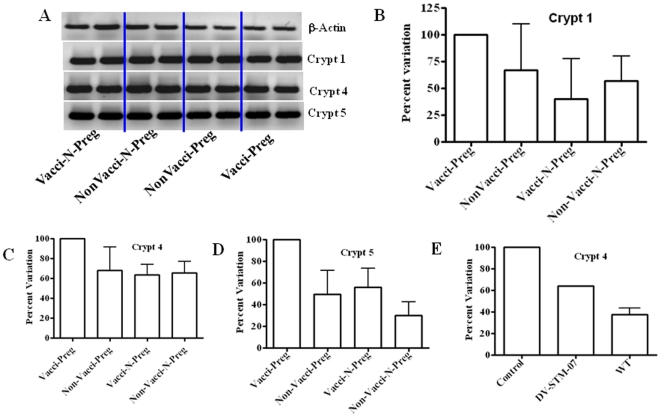
Expression level of Cryptdine in intestine of vaccinated mice is not altered. RT-PCR showing expression level of Cryptdine in intestine of vaccinated; pregnant (Vacci-preg), non-pregnant (Vacci-N-preg) and non-vaccinated; pregnant (NonVacci-preg), non-pregnant (NonVacci-N-preg) mice (A). From each group of mice (Explained in material and methods) intestine was isolated and the RNA isolated using Trizol (SIGMA). RT-PCR was done and densitometric analysis for the expression levels of Cryptdine 1 (B), Cryptdine 4 (C and E) and Cryptdine 5 (D) as against β-actin used as internal control. Data shows that the various cryptdine levels are not down regulated after infection with DV-STM-07 and are similar to control.

The immune reaction against Salmonella involves both the innate and the adaptive immunity. A strong cell mediated immunity though protective can cause abortion by increasing the Th1 cytokines which can lead to fetal rejection. The protection offered by DV-STM-07 against challenge with WT *Salmonella* was surprising as this was achieved without inducing abortion. We suspected that the vaccine strain has a high degree of attenuation which does not elicit a strong inflammatory response but still is able to evoke a protective immune response. WT Salmonella is able to suppress the levels of antimicrobial peptide (AMP) cryptdine in the intestine which helps it to proliferate and spread [30]. AMPs are small cationic peptide with potent bactericidal activity. We looked at the level of cryptdine, in the vaccinated and unvaccinated mice. Small intestine was isolated and RNA was extracted. The RNA was reverse transcribed and semi-quantitative RT-PCR with mouse cryptdine 1, 4 and 5 specific primers was performed. We observed that vaccine strain did not down-regulate the different cryptdine levels as reported for WT *Salmonella*. Moreover, during pregnancy, the levels of all cryptdine tested were more in vaccinated pregnant mice compared to the control pregnant mice though not statistically significant (Figure 1A, B, C and D). The vaccine strain unlike the WT is unable to down regulate the levels of cryptdine ( Figure 1E). This may be one of the reasons why the vaccine strain is easily cleared from the host. Also the host cryptdine may induce lysis of bacteria leading to better antigen presentation.

### Vaccine Strain DV-STM-07 Confers Protective Immunity to the New Born Mice of Vaccinated Mothers

**Figure 2:**
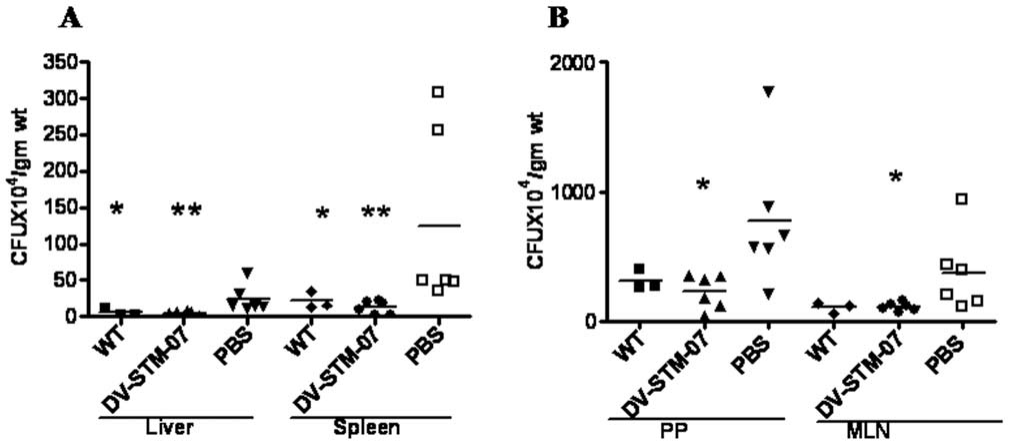
Protective immunity is transmitted to the next generation. Organ CFU was performed on 5^th^ day post infection. Significant decrease in organ CFU was observed in liver & spleen (A) and PP & MLN (B) of pups from vaccinated mother vaccinated with single dose of 10^7^ CFU/mouse during mid pregnancy, than pups from WT and PBS control mothers. (* p<0.05, ** p<0.01, Mann-Whitney test) Data shows the representative of three independent experiments performed.

**Figure 3:**
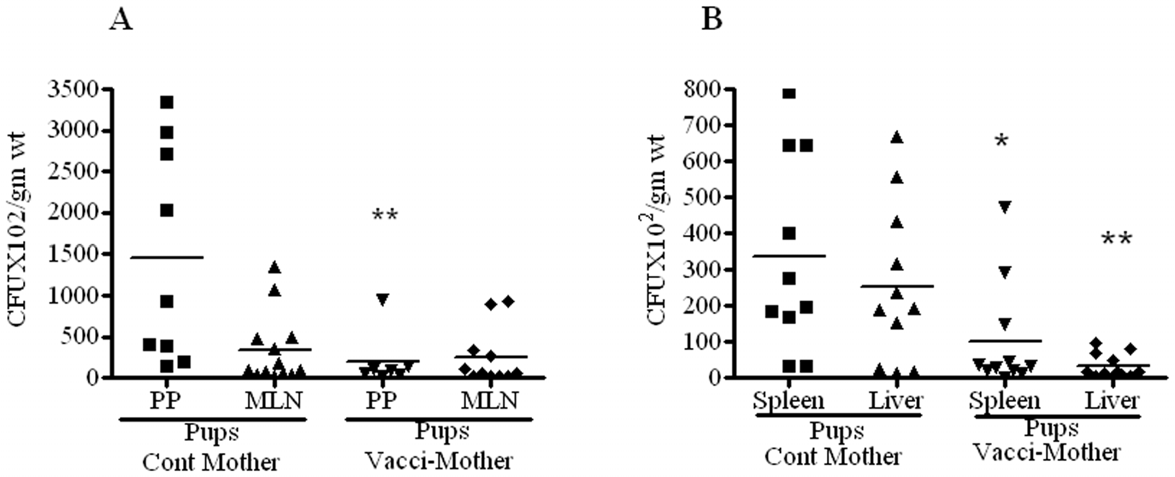
Pre-pregnancy vaccination of mice reduces bacterial burden in pups. Non- pregnant nice were given 3 doses of 10^4^ cfu of vaccine strain at an interval of one week. A week after the last vaccination the mice were mated. The pups of vaccinated and unvaccinated mice were challenged with 10^7^ cfu of WT bacteria and on the 5th day of post infection of pups (4 weeks), organ CFU of Spleen and Liver (A) and PP and MLN (B) was recorded. Data shows the representative of three independent experiments performed. * p<0.05, ** p<0.001 (Mann-Whitney test).

Transmissions of immunity to pups were assessed by measuring the levels of immunity of pups from vaccinated and unvaccinated mothers. The mother received 3 doses of vaccine strain before pregnancy (10^4^ CFU/mice) or one dose (10^7^) during pregnancy. PBS administered, uninfected mice were used as controls. After delivery, when 5–10 days old pups of WT infected mother were sacrificed and checked for organ CFU, very heavy load of bacteria was present in the liver, spleen, PP and MLN of the pup (Table 1). The remaining pups of all the groups were allowed to grow till 4–5 weeks of age and were infected with 10^7^ CFU/mouse of WT *Salmonella*. We observed very high numbers of bacteria in the uninfected PBS control mother's pups and significantly less number of bacteria in the various organs like liver, spleen (Figure 2A), MLN and PP (Figure 2B) of the vaccinated mother's pups. WT infected mother's pups also showed less bacterial load (Figure 2A and B), which could be due to neonatal exposure of pups to *Salmonella* in WT infected mother. To show the vaccine strain is safe and is able to transmit immunity to pups even if administered before pregnancy, we conducted an experiment administering 3 doses of vaccination with 10^4^ CFU per dose given before mating and the pregnancy outcome was recorded (Table 4). The pups of these vaccinated and unvaccinated mothers were challenged at 4 weeks of age with 10^7^ WT bacteria per mouse. The pups were then sacrificed 5 days after challenge and their organs were plated to determine the bacterial load. The CFU in spleen, liver (Figure 3A), PP and MLN (Figure 3B) were lesser than the unvaccinated group. This was found to be statistically significant except in case of MLN. This suggests that immunity is being transferred to the next generation. The possible route of transmission of immunity can be through trans-placental transfer of anti-*Salmonella* IgG or through trans-colostral transfer of antibodies [31]. In another set of experiment the vaccinated and control mother were challenged with 10^7^CFU/mouse of WT *Salmonella* and monitored for pregnancy outcome (Table 2).

**Table 4:** Pregnancy outcome in pre-pregnancy vaccinated mice without WT challenge.

Strain	Dose 10^4^ CFU/mouse	Mating after 1 week	Challenge With WT	Pregnancy Outcome/total pregnant mice			
			Mid pregnancy	Death	Abortion	Resorption	Delivered
DV-STM-07 Vaccine	3 doses	Mated	None	0/4	0/4	0/4	4/4
PBS control	-	Mated	none	0/4	0/4	0/4	4/4

Cohorts of 4 mice were given 3 doses of 10^4^ CFU/mouse of DV-STM-07 at an interval of 7 days. The control mice received PBS. One week after the last dose the mice were mated and observed for the outcome of pregnancy. Experiment was done thrice to reproduce the results.

### Th1 Cytokines INFγ and TNFα Levels Were Significantly Lower in Vaccinated Mice after Wild Type Challenge

**Figure 4:**
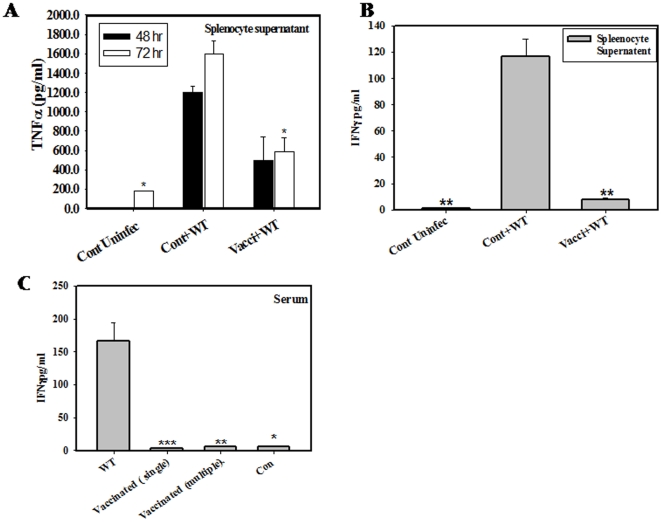
Th1 cytokine levels are lower in the splenocytes of vaccinated mothers. Isolated splenocytes (details in material and methods) were cultured for 48 hrs and 72 hrs. Level of TNFα (A) and INFγ (B) was significantly reduced in primed splenocytes as compared to the control followed by the infection. The level of IFNγ was significantly more in serum of single dose infection with WT *Salmonella* not in control and primed (C). Data shows the representative of three independent experiments performed. * p<0.05, ** p<0.001 *** p<0.0001 (Student “t” test).

**Figure 5:**
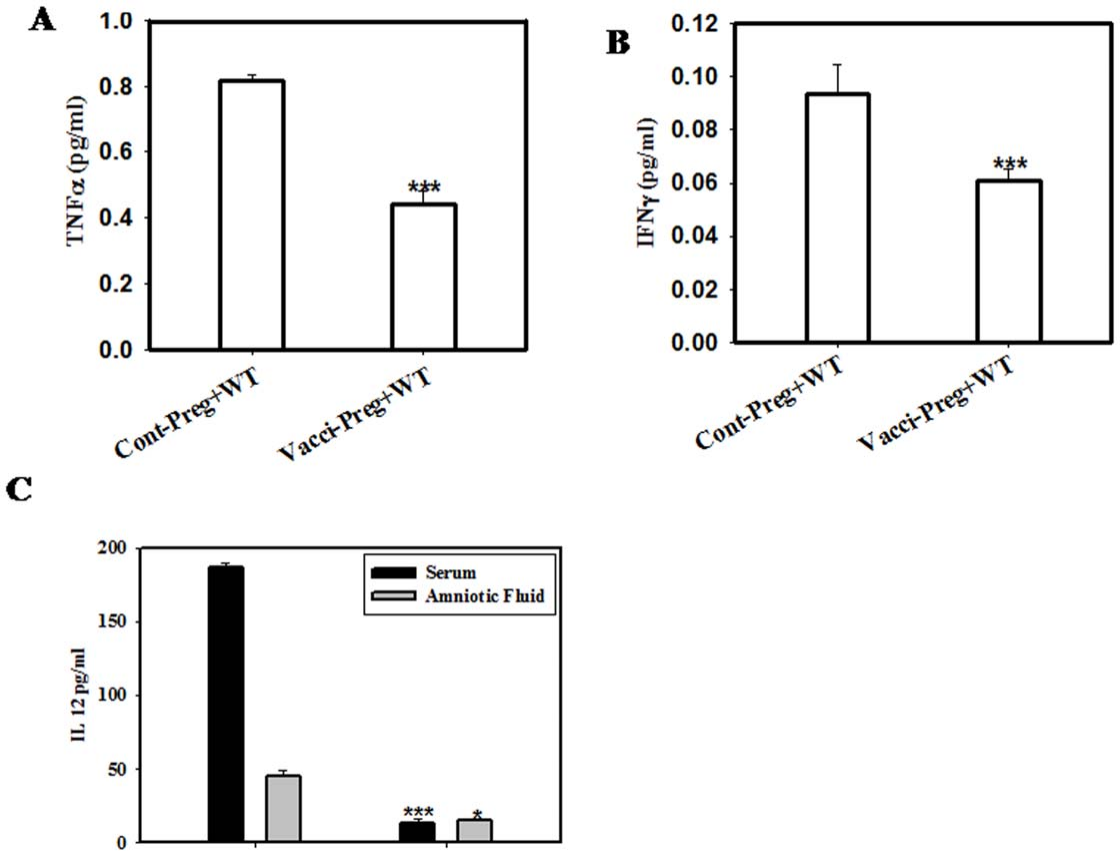
Th1 cytokines were significantly increased in unvaccinated mice as compared to the vaccinated pregnant mice. There is an increase in TNFα (2-fold) in amniotic fluid of unvaccinated mice (A). The serum INFγ levels also increased (B). The IL-12 level in serum as well as in amniotic fluid was higher in unvaccinated group (C). * p<0.05, *** p<0.0001 (Student “t” test).

**Figure 6:**
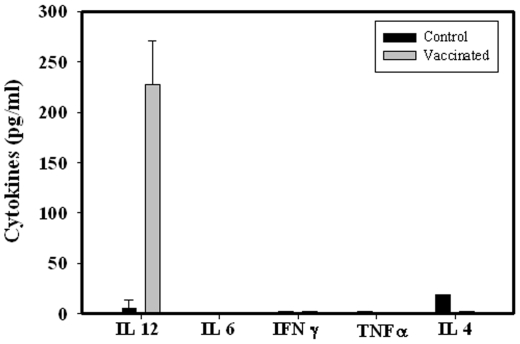
Th1 and 2 cytokine levels in vaccinated than unvaccinated non-pregnant mice. Vaccinated and unvaccinated mice were sacrificed and checked for the cytokine profile. IL-12 was significantly more in vaccinated mice as compared to the unvaccinated mice. There was no difference in other cytokine like IL-6, IL-4, TNFα and IFNγ level in serum of Control and vaccinated mice. Data shows the representative of three independent experiments performed.

During pregnancy, cytokine ratios are altered to facilitate allograft survival. Increase in the cytokines regulating cell mediated immunity classified as Th1 cytokine levels can lead to abortion. The levels of Th1 cytokines; IFNγ and TNFα, following WT challenge in pregnant, vaccinated and unvaccinated mice were analyzed. Splenocytes isolated from pregnant mice (13–15 days of gestation) of both the groups were cultured and cytokine levels in the cell supernatants at different time were measured. The levels of Th1 cytokine TNFα (2–3 fold) (Figure 4A) and IFNγ (11-fold) (Figure 4B) in the cell supernatant were significantly higher in the WT infected pregnant mice as compared to the vaccinated and WT challenged mice which received one priming and two booster doses of vaccine. The level of IFNγ in serum increased drastically after giving single infection with 10^7^ CFU of WT *Salmonella* as compared to the vaccine strain (Figure 4C). The level of TNFα in the amniotic fluid was 1.5 fold more in unvaccinated pregnant infected mice which could be a possible cause of increased incidence of abortion and fetal death in the unvaccinated mice (Figure 5A). IFNγ level were also found to be more in the serum of unvaccinated pregnant mice challenged with WT *Salmonella* than the vaccinated one (Figure 5B). The serum levels of IL-6, IL-4, TNFα and IFNγ were found to be similar in mice given either PBS or DV-STM-07 (Figure 6). Increased Th1 cytokines in pregnant mice infected with WT *Salmonella* indicate the loss of immunosuppressive state of the mother and hence could be deleterious to the fetus. The level of IL-12 was 17-fold less in the serum of vaccinated and challenged pregnant mice as compared to the unvaccinated and challenged pregnant mice. IL-12 level in amniotic fluid of those mice was also 3.5-fold less than control pregnant mice (Figure 5C). But in case of non pregnant mice, the level of IL-12 was more after vaccination as compared to the control mice (Figure 6). The cytokine profile observed was pregnancy specific which differs from the normal non pregnancy condition of the mice.

### Th2 Cytokine Profile in Mice with Priming and Infection

**Figure 7:**
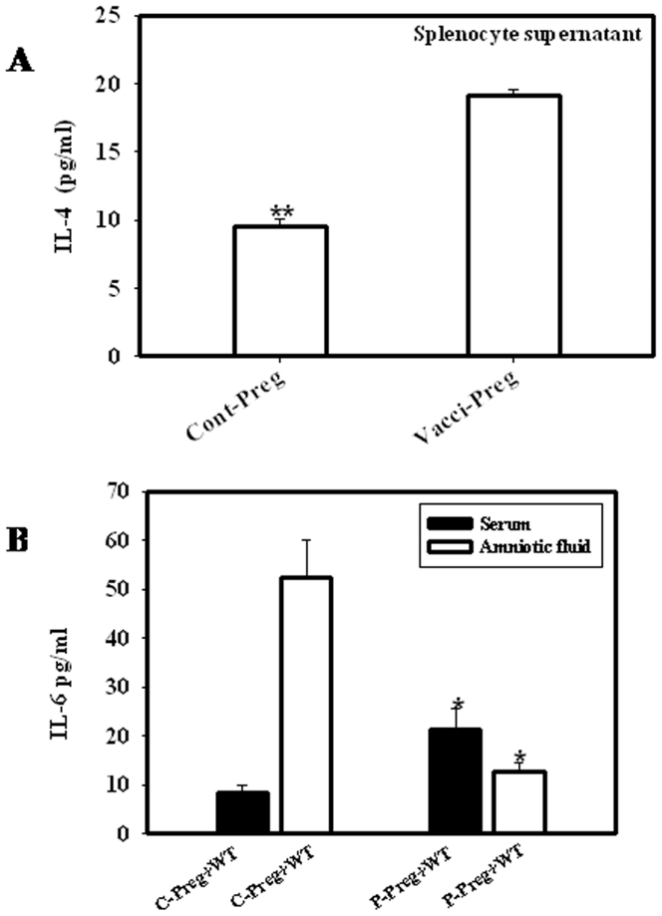
Th2 cytokine levels were higher in vaccinated than unvaccinated pregnant mice. Vaccinated and unvaccinated mice were sacrificed and checked for the cytokine profile. IL-4 was significantly more in vaccinated mice as compared to the unvaccinated mice (A) Levels of Serum IL-6 was enhanced in vaccinated mice but reverse was observed in case of the amniotic fluid of vaccinated and unvaccinated mice (B). Data shows the representative of three independent experiments performed. * p<0.05, ** p<0.001(Student “t” test).

The cytokines regulating humoral immunity classified as Th2 cytokine, IL-4 signaling is very important for the development of plasma cells from pre B cells which is the most important step in the development of an effective humoral immunity. IL-4 also suppresses the development of Th1 mediated immune response. Hence, we checked the level of IL-4 secreted by the lymphocytes following vaccination. The splenocytes were isolated from vaccinated and unvaccinated pregnant mice and were maintained for 72 hrs in culture. The IL-4 levels were measured from the culture supernatants. Th2 cytokine IL-4 was increased almost by 2-folds in vaccinated pregnant mice (Figure 7A). We further analyzed the level of another Th2 cytokine IL-6, which plays a crucial role in immune regulation during pregnancy. Following vaccination, the IL-6 levels were determined in the serum of the mother and the amniotic fluid. IL-6 showed a contrasting profile in the serum and the amniotic fluid compartments. With vaccination, IL-6 level in the serum increased by 2-fold but the increase was not statistically significant. Level of IL-6 in amniotic fluid was 4-fold less in vaccinated pregnant mice as compared to the unvaccinated pregnant mice (Figure 7B). This drop is significant in preventing abortion, as IL-6 release stimulates prostaglandin synthesis by uterine tissues [32], [33]. The increase in the IL-4 and IL-6 levels in the maternal serum shows that the immune response has been biased towards the Th2 immune response. The drop in the IL-6 levels in the amniotic fluid also reveals that these levels do not follow the serum cytokine levels. But the overall change in the Th2 cytokine in maternal serum indicates the development of a fetus protecting cytokine milieu in the amniotic fluid compartment. Th2 serum cytokine profile also was not altered in the non-pregnant mice upon vaccination (Figure 6).

## Discussion

Pregnancy increases the incidence of many bacterial and viral diseases by lowering the immunity. This increased susceptibility leads to infection induced abortion or congenital defects in the fetus. Pregnancy is a condition where the immune system is faced with the situation of not mounting an immune reaction against the foreign paternal antigens of the fetus, at the same time should protect self and the fetus from invading pathogens. The immune system overcomes this dilemma by altering the Th1/Th2 cytokine levels to favor Th2 cytokines. This protects the fetus from the Th1 mediated immune rejection at the fetal-maternal interface but at the same time makes pregnant women susceptible to a host of infection where the immunity is Th1 dependent like in malaria [14], leishmaniasis [13], toxoplasmosis, leptospirosis [15] and salmonellosis [19]. *Salmonella* infection among other infections has the ability to induce abortion [4], [34] Prophylactic vaccination is an effective way to prevent many of the diseases that are a risk factor for pregnant women. Our previous study showed that DV-STM-07 (Δ*pmrG-HM-D*) mutant vaccine strain [27] is better than *aroA* [35], [36] mutant vaccine strain in terms of lower and lesser number of doses and better protection. Our current study shows that vaccination with live attenuated S. Typhimurium vaccine DV-STM-07 [27] protects pregnant mice from abortion, prevents fetal death and decreases the rate of resorption in vaccinated mice. DV-STM-07 vaccination was able to reduce the bacterial load significantly in mice after challenge with WT *Salmonella* [27].

The immunity against *Salmonella* involves both the Th1 regulated cell mediated immunity [37], [38] and the Th2 regulated humoral immunity [39], [40]. This is unlike a few intracellular parasites where protection is conferred only by cell mediated immunity like in the case of malaria [14]. Since both arm of the adaptive immune system are effective against *Salmonella*, our vaccine candidate is able to exploit the Th2 dominated environment of pregnancy and is able to impart protection. We found that Th1 cytokines (IFNγ, TNFα, IL-12) were significantly high in cell supernatant of splenocytes and also in the serum of unvaccinated and challenged mice as compared to the vaccinated mice. This scenario was also reflected in the amniotic fluid of the mice in case of IFNγ and TNFα. Previous studies have shown that the basal serum and amniotic fluid levels of IFNγ are very low and their level increases in amniotic fluid only nearing term which may signal parturition [41], [42]. The serum IFNγ, IL-12 and IL-18 levels were increased significantly in patients with both systemic and gastrointestinal form of salmonellosis [43]. IL-12 has been known to play an important role in *Salmonella* infection. In humans, IL-12 deficiency increases susceptibility to *Salmonella* infection [44]. Strikingly, the opposite is true for pregnancy. High IL-12 expression is very deleterious during pregnancy[45]. Hence it makes sense that DV-STM-07 vaccination led to decrease in the IL-12 levels in WT challenged mice as opposed to the high level of IL-12 in unvaccinated and challenged mice. The net outcome was the protection of the pregnant mice against abortion. The cytokine profile of the mother is independent of cytokine profile of amniotic compartment and level of cytokine in mother's circulation does not reflect the profile in the amniotic regions [46], [47]. But the level of cytokines in both the compartments have significant role in the outcome of pregnancy. The low level of Th1 cytokine in vaccinated mice indicates that the immune system is able to contain the infection unlike the unvaccinated mice where the high levels of these cytokines can induce abortion [29]. The reduced Th1 cytokines were also coupled with the increased Th2 cytokine especially IL-4. Another Th2 cytokine, IL-6 has a more intricate role in immune regulation of pregnancy. IL-6 level was found to be very low in the amniotic fluid and fetal serum of mid-term pregnant women [48] but it increases near term and during intrauterine infection and signals parturition [32], [33]. IL-6 in the maternal compartment is also a crucial regulator of humoral immune response [49]. There is a significant drop in the IL-6 levels in the amniotic fluid and an increase though non-significant in the maternal serum levels in the vaccinated compared to the unvaccinated mice. This explains the effective Th2 mediated immune protection for which IL-6 is needed in the maternal serum and also the low IL-6 levels in the amniotic fluid helps in preventing abortion.

The vaccine strain DV-STM-07 is able to achieve protective immunity without triggering abortion because of its high degree of attenuation and also its ability to not induce inflammation. WT *Salmonella* on the other hand induces severe inflammation after infection which is accompanied by increase in the pro-inflammatory cytokines of Th1 arm [43]. WT *Salmonella* are able to down regulate the AMPs which is an important evasion strategy. The safety of vaccination with our vaccine strain in pregnant mice may be due to the unaltered levels of cryptdine. Cryptdine is one of the important AMP which is known to have anti-salmonella activity. Hence, cryptdine might have helped to clear the bacteria from the vaccinated mice and also resulted in efficient antigen presentation. Apart from protecting the mice from abortion, this vaccine also conferred immunity to the pups of the vaccinated mother. The transmission of the vaccine strain to the pups through transplacental route or during birth has been ruled out. This suggests that the transfer of immunity is through passive transfer of colostral antibodies which has previously been shown for sheep, which received live attenuated *Salmonella abortisovis* strain during pregnancy [31]. We have shown in our previous study that vaccine strain increase CD8^+^ T cells response in vaccinated mice [27]. However, during pregnancy we could not detect any appreciable difference in the CD4+ and CD8+ T cell population (Data not shown). This could be due to the altered immune response during pregnancy which suppresses cell mediated immunity which is reflected in the lack of expansion of the CD8+ T cell compartment. This study is the first of its kind which addresses a very important issue of abortion induced by *Salmonella*. Our vaccine strain prevented the abortion and conferred immunity to the pups. This has an important healthcare application, as till to date no vaccine is there in the market against typhoid which can be delivered to the pregnant women. The loss of pregnancy by salmonellosis is poorly documented. However, our research shows a high risk of abortion in pregnant mice model and hence this open up further avenues for deeper thoughts in vaccine development.

## Materials and Methods

### Bacterial Strains, Media and Growth Conditions

Bacterial strains used in this study are derived from wild type *S. enterica serovar* Typhimurium strain 12023 (kind gift by Prof. M.Hensel). Vaccine strain *pmrG-HM-D* (DV-STM-07) was from our previous study [27]. Bacteria were grown at 37°C in Luria broth (LB) WT and the vaccine strain were grown in respective selective antibiotic at the concentration of 50 µg/ml of carbenicillin and 20 µg/ml of chloramphenicol. Bacteria were diluted in phosphate buffered saline (PBS) and used for vaccination and challenge of mice with desired CFU.

### Mice Immunization, Mating and Infection Method

Immunization was carried out as described earlier [27]. All the procedures with animals were carried out in accordance with the approved protocols of Indian Institute of Science. Cohort of 25 BALB/c female mice of five to six weeks old were immunized with DV-STM-07 vaccine strain with 10^4^ CFU/mouse followed by two booster dose at day 7 and 14. After 7 days of second booster dose mice were kept for mating with 8–9 week old isogenic BALB/c male for two days to increase the percentage of pregnant mice and subsequent day after checking the vaginal plug was counted as day 1 of pregnancy. Equal numbers of mice were given only PBS and were used as controls in all the experiments performed. Mice were monitored for pregnancy outcome and cytokines profiles were checked as described in material and methods section. 6–7 pregnant mice from each vaccinated and unvaccinated group were given WT *Salmonella* challenge with 10^7^ CFU/mouse during mid pregnancy (13–15days pregnant) and monitored everyday. After a gestation period of 21 days, the mice were put under constant surveillance for signs of parturition. Non pregnant (10–20% of total kept for mating) mice also were given lethal *Salmonella* challenge and observed for survivability.

Uninfected pregnant mice were also observed similarly. Offspring were allowed to grow for 4 weeks and were infected with 10^7^ CFU/mouse. Organ load of bacteria was checked in spleen, liver, Peyer's patches (PP)and mesenteric lymph nodes (MLN) of those now adult, weaned pups.

### RNA Isolation and RT-PCR from Tissue

From the group of vaccinated and non-vaccinated, pregnant and non-pregnant mice of each group, distal part of intestine was taken and frozen in LN2. The frozen tissue was crushed to powder in mortar and pestle and 1 ml of TriZol reagent (SIGMA) was added and processed further according to the manufacturer's instruction. RNA quality was checked by running gel, quantified in ND 1000 spectrophotometer (Nanodrop) and stored in −70°C till further use. One µg of DNase treated RNA was heated at 70°C for 10 min to remove secondary structure and then cooled on ice. Reverse transcription of the RNA was performed by using RT-kit (Promega) as per manufacturer's protocol. cDNA obtained was checked for the mRNA levels of cryptidin1, 4 and 5 with mouse β-actin mRNA levels as internal control. List of primers are given in table 5.

**Table 5:** List of primers used for semi-quantitative RT-PCR of cryptdine levels.

Primers	Sequence 5′ - 3′
*Crypt1* forward	AAGAGACTAAAACTGAGGAGCAGC
*Crypt1* reverse	GGTGGTCATCAGGCACCAGCATCAGT
*Crypt4* forward	AAGAGACTAAAACTGAGGAGCAGC
*Crypt4* reverse	AGTGGTCATCAGGCCCCGGCATCAGC
*Crypt5* forward	AAGAGACTAAAACTGAGGAGCAGC
*Crypt5* reverse.	GGTGATCATCAGACCCCAGCATCAGT
*β actin* forward	TGG AATCCT GTGGCATCCA
*β actin* reverse	TAACAGTCCGCCTAGAAGCA

### Cytokine Assay

Cohort of Uninfected pregnant mice and WT *Salmonella* infected pregnant mice were sacrificed at different time points. Splenocytes were isolated from naive and immunized mice and pregnant and non pregnant mice within the respective groups. After RBC lysis, 2×10^6^ cells/well was seeded with or without PMA (phorbol 12-myristate-13-acetate, SIGMA), a non specific stimulator of T cells in RPMI medium supplemented with 10% FBS, 50 µg/ml of penicillin and streptomycin (Sigma). Cells were incubated at 37°C/5% CO_2_ and supernatants were collected after 24, 48 and 72 hrs post incubation. Supernatants were stored at −20°C till further use. Serum and amniotic fluid were also collected from infected and uninfected naïve and vaccinated pregnant mice and stored at −20°C for further use. Serum from non-pregnant vaccinated and non vaccinated mice was collected for the cytokine analysis.

### Enzyme Linked Immunosorbent Assay (ELISA)

ELISA was performed for different cytokines like TNFα, IFNγ, IL-4 (BD Bioscience) and IL-6, IL-12 (eBioscience) in serum, splenocyte supernatant and amniotic fluid from the mice of each group described before. Assay was performed as per the instruction of manufacturer. Data was analyzed by using Graph Pad Prism and statistical analysis was done for all the assays performed using Mann-Whitney and Student's t-test.

### Statistical Analysis

The data were subjected to statistical analysis by applying Student's *t*-test for cytokine levels and Mann–Whitney test for organ cfu load by using Sigma plot and Graph Pad prism 4 softwares respectively. The mortality and survival data were analyzed using Fisher's exact test. *p*-values of <0.05was considered significant.
